# Novel Loss-of-Function Mutations in *DNAH1* Displayed Different Phenotypic Spectrum in Humans and Mice

**DOI:** 10.3389/fendo.2021.765639

**Published:** 2021-11-17

**Authors:** Ranjha Khan, Qumar Zaman, Jing Chen, Manan Khan, Ao Ma, Jianteng Zhou, Beibei Zhang, Asim Ali, Muhammad Naeem, Muhammad Zubair, Daren Zhao, Wasim Shah, Mazhar Khan, Yuanwei Zhang, Bo Xu, Huan Zhang, Qinghua Shi

**Affiliations:** ^1^ First Affiliated Hospital of University of Science and Technology of China (USTC), Hefei National Laboratory for Physical Sciences at Microscale, School of Basic Medical Sciences, Division of Life Sciences and Medicine, Chinese Academy of Sciences (CAS) Center for Excellence in Molecular Cell Science, University of Science and Technology of China, Hefei, China; ^2^ Medical Genetics Research Laboratory, Department of Biotechnology, Quaid-i-Azam University, Islamabad, Pakistan; ^3^ Reproductive and Genetic Hospital, The First Affiliated Hospital of University of Science and Technology of China (USTC), Division of Life Sciences and Medicine, University of Science and Technology of China, Hefei, China

**Keywords:** MMAF, male infertility, mutant mice, *DNAH1*, central singlet

## Abstract

Male infertility is a prevalent disorder distressing an estimated 70 million people worldwide. Despite continued progress in understanding the causes of male infertility, idiopathic sperm abnormalities such as multiple morphological abnormalities of sperm flagella (MMAF) still account for about 30% of male infertility. Recurrent mutations in *DNAH1* have been reported to cause MMAF in various populations, but the underlying mechanism is still poorly explored. This study investigated the MMAF phenotype of two extended consanguineous Pakistani families without manifesting primary ciliary dyskinesia symptoms. The transmission electron microscopy analysis of cross-sections of microtubule doublets revealed a missing central singlet of microtubules and a disorganized fibrous sheath. SPAG6 staining, a marker generally used to check the integration of microtubules of central pair, further confirmed the disruption of central pair in the spermatozoa of patients. Thus, whole-exome sequencing (WES) was performed, and WES analysis identified two novel mutations in the *DNAH1* gene that were recessively co-segregating with MMAF phenotype in both families. To mechanistically study the impact of identified mutation, we generated *Dnah1* mice models to confirm the *in vivo* effects of identified mutations. Though *Dnah1* ^△iso1/△iso1^ mutant mice represented MMAF phenotype, no significant defects were observed in the ultrastructure of mutant mice spermatozoa. Interestingly, we found DNAH1 isoform2 in *Dnah1* ^△iso1/△iso1^ mutant mice that may be mediating the formation of normal ultrastructure in the absence of full-length protein. Altogether we are first reporting the possible explanation of inconsistency between mouse and human *DNAH1* mutant phenotypes, which will pave the way for further understanding of the underlying pathophysiological mechanism of MMAF.

## Introduction

Male infertility is a global health concern with social and psychological impact on more than 70 million infertile men (1). Recent studies have demonstrated that sperm defects, such as reduced sperm count, decreased sperm motility, and abnormal morphology, are directly linked with male infertility (2). Sperm motility is required for normal fertilization, and around 80% of infertility is caused by compromised sperm motility (3). Subsequently, sperm morphology is also an important player necessary for sperm locomotion, and defects in sperm morphologies are associated with a major form of male infertility (4). Defects in sperm morphology are generally manifested with a wide range of phenotypic variations in the head, neck, mid-piece, and tail of spermatozoa. Among these, the most common type of abnormities is found in the form of multiple morphological abnormalities of sperm flagella (MMAF) in asthenospermia patients. The most common defects of sperm flagella observed in MMAF phenotype include coiled, bent, irregular, short, absent, or irregular-width flagella (5). Normal sperm flagella contain a typical axonemal arrangement of nine peripheral microtubules and two central microtubules (CP), which are further surrounded by dense outer fiber and fibrous sheath (6). This basic structure of flagella remains conserved through the process of evolution. Any structural aberrations in these components of sperm flagella are associated with a wide range of MMAF phenotypes which further caused a reduction of sperm motility or even sperm immobility and eventually male infertility.

*DNAH1* (MIM 603332) is an essential gene that encodes inner dynein arm (IDA) heavy chain, which are believed to strengthen the link between radial spokes and outer doublet (7). IDA, an ATPase-based protein complex, is responsible for beat generation and regulation of flagellar movement (8, 9). Recurrent mutations in *DNAH1* are commonly associated with MMAF in various populations (7, 10, 11). Moreover, researchers are extensively investigating the molecular mechanism of MMAF by generating knockout (KO) mice models of various genes that have been associated with MMAF phenotype in humans (6). Until now, mutations in various genes, such as *CFAP43*, *CFAP44*, *CFAP47*, *CFAP58*, *CFAP65*, *CFAP69*, *CFAP70*, *CFAP74*, *CFAP91*, *CFAP206*, *CFAP251*, *DNAH1*, *DNAH2*, *DNAH6*, *DNAH8*, *DNAH10*, *DNAH17*, *WDR19*, *ARMC2*, *TTC21A*, *TTC29*, *FSIP2*, *AK7*, *CEP135*, *SPEF2*, *QRICH2*, *DZIP1*, *BRWD1*, *DRC1*, *STK33*, *etc.*, have been reported with MMAF phenotype (10, 12–25). However, a discrepancy exists in the *Dnah1* KO mouse model and human phenotype (26, 27). A previous study demonstrated that *Dnah1* KO mice are completely infertile due to defects in sperm motility, but the ultrastructure of flagella did not show any abnormalities in their components (26). On the other hand, *DNAH1* mutant human sperm ultrastructure was severely compromised because of lacking CP and other defects. Thus, uncovering the inconsistency between human and mouse phenotypes of the *DNAH1* gene required a deeper study to discover the unknown isoforms mediating the intact formation of normal ultrastructure of mouse sperm flagella in the absence of full-length DNAH1 protein.

In the present study, we recruited two extended consanguineous Pakistani families suffering from male infertility due to the MMAF phenotype. Subsequently, whole-exome sequencing (WES) identified two novel mutations in the *DNAH1* gene that were recessively co-segregating with infertility phenotype in both families. Furthermore, we generated *Dnah1* mice models to confirm the *in vivo* effects of identified mutations. Interestingly, we found *Dnah1* isoform2 in *Dnah1* ^△iso1/△iso1^ mutant mice that may be mediating the formation of normal ultrastructure in the absence of full-length protein. Altogether we are the first one to present the possible explanation of inconsistency between mouse and human *DNAH1* mutant phenotype, which will pave the way for further understanding of the underlying pathophysiological mechanism of MMAF.

## Materials and Methods

### Recruitment of Families and Phenotype Confirmation

Two consanguineous families from Pakistan, comprising six male patients, were enrolled to investigate the underlying genetic cause of male infertility. Detailed pedigrees were constructed according to the provided information by the parents of the patients. An initial physical and andrological examination displayed that all patients have normal body mass index and no primary ciliary dyskinesia (PCD) symptoms. Other associated diseases, such as hypogonadotropic hypogonadism, cryptorchidism, varicocele, seminal ductal obstruction, testicular trauma, and andrological tumor, were also not observed. Two repeated semen analyses were performed according to the WHO manual of 2010, and all the parameters were recorded (**Table 1**). We also recruited the fertile siblings of patients and their parents to serve as a positive control for genetic analysis. The study was approved by a bioethical committee of Pakistan and the ethical review board of the University of Science and Technology of China. Informed consent was obtained from all participants of both families.

**Table 1:** Clinical characteristics of patients.

	Reference values	PK-INF-15	PK-INF-319		
		IV:1	V:1	V:2	V:3
Gene	–	*DNAH1*	*DNAH1*	*DNAH1*	*DNAH1*
cDNA mutation	–	c.7646_7647InsC	c.6212T>G	c.6212T>G	c.6212T>G
Protein alteration	–	p.N2549Qfs*61	p.C1789Y	p.C1789Y	p.C1789Y
Fertility	–	Infertile	Infertile	Infertile	Infertile
Age (years old)^a^	–	1978	1980	1984	1986
Years of marriage	–	2003	2005	2007	2010
Height/weight (cm/kg)	–	183.0/70.0	183.0/99.0	180.0/86.0	180.0/86.0
Semen parameters^b^					
Semen volume (ml)	>1.5	1.5	2.4 ± 0.4	2.5 ± 0.5	3.8 ± 0.8
Semen pH	Alkaline	Alkaline	Alkaline	Alkaline	Alkaline
Sperm concentration (10^6^/ml)	>15	6.0	3.0 ± 0.5	7.0 ± 4.0	20.0 ± 5.0
Normal sperm morphology (%)	>4	0	2.6	0.6	–
Motile sperm (%)	>40	0	0	0.5 ± 0.5	0
Progressively motile sperm (%)	>32	0	0	0	0
Sperm flagella					
Morphologically normal (%)	–	0	2.6	0.6	–
Absent (%)	–	11.0	24.7	26.8	–
Short (%)	–	22.0	24.3	26.2	–
Coiled (%)	–	19.0	30.5	29.2	–
Bent (%)	–	5.0	4.8	8.0	–
Irregular caliber (%)	–	43.0	13.1	9.2	–

^a^Ages at the manuscript submission. ^b^Reference values were published by WHO in 2010.

### WES and Linkage Analysis

Genomic DNA was extracted from all available members of both families by using QIAamp Blood DNA Mini Kit (QIAGEN) as per the protocol of the manufacturer. WES was conducted on III:2, IV:1, III:3, V:1, V:2, V:3, and V:5 from family members of PK-INF-15 and PK-INF-319, respectively, previously reported in (28). Briefly, WES data from the selected members were enriched by the Agilent SureSelect XT Human All Exon Kit. The Illumina HiSeq XTEN platform accompanied next-generation sequencing. The obtained raw reads were aligned to the human genome reference assembly (GRCh37/hg19) using the Burrows-Wheeler Aligner (29). Next, the Picard software was engaged to remove polymerase chain reaction (PCR) duplicates and evaluate the quality of variants. DNA sequence variants were analyzed by the best practice genome analysis kit (30). VCF files were used for parametric linkage analysis, as described previously (31), and four linkage regions were identified with a logarithm of odds (LOD) score of more than 0.5. Further screening was opted for the variants that were located on linkage regions and following Mendelian inheritance pattern.

### Filtering and Selection of Candidate Variants

Only variants that have depth >×20, genotype quality >90, and 0.5-cM intervals between each other were designated as markers. MERLIN software was employed on genotyped single-nucleotide polymorphism for linkage analysis with these parameters: due to consanguinity in the families, recessive mode of inheritance was adopted, with a disease allele frequency of 0.001 and 100% penetrance. A number of five peaks with a LOD score of more than 0.5 were considered as linkage regions. The variants that resided in the linkage regions were further annotated by ANNOVAR using the NCBI RefSeq gene annotation. We adopted the following filtration strategy to select potential candidate disease-causing variants in patients: (i) variants that were heterozygous in the father and control brothers and homozygous in patients were retained; (ii) variants with minor allele frequency of more than 0.01 in public databases such as 1000 Genome project (32), ESP6500 (33), or ExAC database (34), and variants homozygous in our in-house WES variants call set generated from 578 fertile male samples (41 Pakistanis, 254 Chinese, and 283 Europeans) were excluded; (iii) variants that affect protein sequence and predicted to be deleterious by around 10 of 13 *in silico* employed tools were kept; and (iv) variants which have a predominant expression in the testes and their inactivation affects male fertility were retained based on spermatogenesis online database (35) and were checked by Sanger sequencing to verify Mendel inheritance pattern. All these strategies are more clearly explained in a flow chart diagram in **Supplementary Figure S2**. Finally, only *DNAH1* variants followed the inheritance pattern and thus were considered the potential candidates causing MMAF phenotype in subjects under investigation. The list of primers used for Sanger sequencing is shown in **Supplementary Table S1**.

### Immunofluorescence Staining

Immunofluorescence on the spermatozoa of patients was performed, as we have previously reported (36). Briefly, sperm from patients were smeared on the clean slide and fixed with 4% paraformaldehyde followed by three times of washing in phosphate-buffered saline (PBS). The slides were permeabilized with 0.5% Triton X-100 for 30 min and blocked with 1% BSA. Next, the slides were incubated with primary antibodies, including α-tubulin (Sigma, F2168), DNAH1 (Abcam, ab122367), and SPAG6 (Proteintech, 12462-1-AP), overnight at 4°C. On the next day, the slides were washed with PBST (PBS containing Triton X-100) and incubated with secondary antibodies DAR555 (Molecular Probes, A31572) and GAM488 (Molecular Probes, A21121) for 1 h at 37°C. Finally, the slides were washed again and sealed with Hoechst and Vectashield. Images were captured by using a laser scanning confocal microscope (Olympus).

### Electron Microscopy Analysis

Sperm samples from patients were processed in 0.1 M phosphate buffer (pH 7.4) containing 4% paraformaldehyde, 8% glutaraldehyde, and 0.2% picric acid at 4°C. Scanning and transmission electron microscopy (SEM and TEM) were performed as previously described, with minor modifications (36). Briefly, spermatozoa were washed in 0.1 M PBS four times, followed by fixation in 1% OsO_4_ and dehydration. Then, samples were infiltrated for acetone and epon resin mixture and embedded in sputter-coated (SCD 500, Bal-Tel). Thinly sectioned (70 mm) samples were stained with uranyl acetate and lead citrate. The morphology of spermatozoa was examined by Hitachi S-4800 field emission scanning electron microscope under an accelerating voltage of 15 kV. The ultrastructure of the samples was analyzed and captured by Tecnai 10 or 12Microscope (Philips) at 100 or 120 kV or by H-7650 microscope (Hitachi) at 100 kV.

### Generation of Mutant Mice Models

*Dnah1^-/-^* and *Dnah1^△iso1/△iso1^* mice were generated by using CRISPR/Cas9 genome editing technology as previously reported (37). Briefly, single guide RNAs (sgRNAs) designed to target exon 2 and 49 of *Dnah1* to generate KO and mutant mice, respectively, were transcribed *in vitro* (Addgene, 5132). Single-strand oligodeoxynucleotides (ssODNs), with a mutation similar to family A2 patients and a synonymous mutation at the protospacer adjacent motif, were created by the Sangon Biotech system. The designed sgRNAs and ssODNs were microinjected with Cas9 mRNAs into the zygotes of B6D2F1 (C57BL/6×DBA/2J) mice, following a previously published methodology (38). The genotyping of newborn pups was determined by Sanger sequencing, and heterozygous Dnah1+/- and Dnah1+/△iso1 mice were bred to obtain the desired homozygous results of *Dnah1^-/-^* and *Dnah1^△iso1/△iso1^* mice for future experiments. The sequence of guide RNAs and all the primers used for genotyping is shown in **Supplementary Table S1**.

### Western Blotting

Testes from 8-week-old *Dnah1^-/-^* and *Dnah1^△iso1/△iso1^* mice were detached and processed in radio-immunoprecipitation assay buffer (25 mM Tris-HCl, pH 7.6, 150 mM NaCl, 1% NP-40, 1% sodium deoxycholate, and 0.1% sodium dodecyl sulfate) complemented with protease inhibitors cocktail using a Tissuelyzer and then cleared by centrifugation. Western blotting was performed as previously reported (39). Nitrocellulose membranes were incubated with primary antibodies against DNAH1 (this study) and GAPDH overnight at 4°C. On the next day, membranes were washed in PBST and incubated with secondary antibodies for 1 h at room temperature. The blots were developed with chemiluminescence (Image Quant LAS 4000, GE Healthcare).

### RNA Extraction and Reverse Transcriptase-PCR

According to the protocol of the manufacturer, total RNA from mice and control human testes was extracted by using Trizol (Invitrogen) kit. RT-PCR was conducted as we have previously described (40). The primer used for RT-PCR is listed in **Supplementary Table S1**.

### Phenotypic Investigation of Mutant Mice

The fertility status of *Dnah1^△iso1/△iso1^* mice was assessed by caging each mutant mouse with two wild-type females (C57/BL) for 3 months. A total of five *Dnah1^△iso1/△iso1^* mice were tested, and none of them produce a single pup (**Table 2**). Testes morphology, sperm count, H&E, sperm morphology, and sperm motility were performed as previously described (36, 39).

**Table 2:** Characteristics of *Dnah1^+/△iso1^*, *Dnah1^△iso1/△iso1^*, *Dnah1^+/-^*, and *Dnah1^-/-^* male mice.

	*Dnah1^+/△iso1^*	*Dnah1^△iso1/△iso1^*	*Dnah1^+/-^*	*Dnah1^-/-^*
Body weight (g)	24.87 ± 2.91	23.11 ± 2.63	19.28 ± 1.92	19.68 ± 4.32
Testis weight (mg)	188.1 ± 26.70	171.0 ± 17.70	128.5 ± 14.10	137.9 ± 36.8
Testis/body weight ratio (10^-3^)	7.56 ± 0.64	7.48 ± 1.27	6.77 ± 1.18	7.03 ± 0.95
Semen parameters				
Sperm count (10^7^)	1.17 ± 0.15	0.45 ± 0.08	1.05 ± 0.12	0.51 ± 0.03
Motile sperm (%)	71.10 ± 7.08	26.53 ± 7.03	70.74	39.56
Progressively motile sperm (%)	43.12 ± 1.13	3.97 ± 2.11	54.59	10.67
Sperm flagella				
Normal (%)	81.84 ± 1.33	33.95 ± 7.58	84.01 ± 1.35	45.46 ± 5.91
Absent (%)	16.13 ± 0.73	53.11 ± 9.63	12.20 ± 2.67	48.56 ± 7.18
Short (%)	0.56 ± 0.19	3.68 ± 1.12	0.72 ± 0.32	1.12 ± 0.37
Coiled (%)	0.31 ± 0.28	2.37 ± 0.67	0.13 ± 0.13	1.19 ± 0.01
Bent (%)	0.98 ± 0.56	1.45 ± 0.69	2.50 ± 1.16	1.87 ± 1.14
Irregular (%)	0.19 ± 0.19	5.44 ± 1.30	0.43 ± 0.26	1.79 ± 0.66

For semen analysis, three 8-week-old mice were examined for each genotype. Data are presented as mean ± SEM.

## Results

### Recruitment of Infertile Patients Manifesting MMAF Phenotype

**Figure 1:**
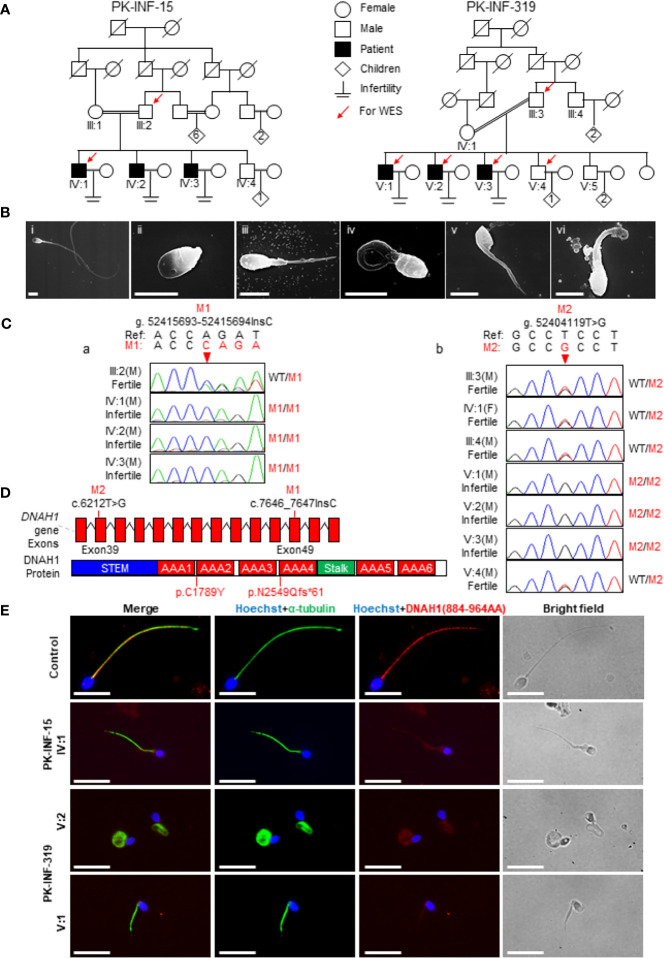
Recruitment of infertile families displaying multiple morphological abnormalities of sperm flagella phenotype. **(A)** Two consanguineous Pakistani infertile families (PK-INF-15 and PK-INF-319), comprising six male patients, were recruited. Male individuals are represented by squares, while female individuals were indicated with a circle. Diamonds indicate offspring, and the numerals inside designate the number of offspring. Solid squares are used to indicate patients, slashes represent deceased family members, and parallel slash lines specify consanguineous marriages. The red arrows indicate the members selected for whole-exome sequencing. **(B)** Scanning electron microscopy of sperm control (i) and from patients [PK-INF-319], V:1 (ii, ii, and iv) and V:2 (v and vi)] displayed various defects of flagella, including absent, short, coiled, and irregular calibers. Scale bar, 10 um. **(C)** Chromatograms representing the segregation of *DNAH1* mutations in available members from families (a) PK-INF-15 and (b) PK-INF-319. The red arrows show the genomic position of *DNAH1* mutations. **(D)** An illustrative representation of DNAH1 gene and subsequent protein structure showing the identified mutation positions. **(E)** No DNAH1 signals were found on paraformaldehyde-fixed sperm from a patient stained against DNAH1 and α-tubulin, while intact DNAH1 signals were observed on sperm obtained from a healthy control. Scale bar, 10 um.

In the present study, we recruited two infertile consanguineous Pakistani families (PK-INF-15 and PK-INF-319) consisting of six patients that were suffering from primary infertility (**Figure 1A**). The semen analysis of four patients showed a sufficient sperm concentration, while all sperms were immotile (**Table 1**). SEM and light microscopy investigation revealed abnormalities in sperm tail, displaying different defects of sperm flagella, including absent, short, coiled, bent, and irregular caliber, suggesting MMAF (**Supplementary Figures S1A, B**). Similarly, the SEM of sperm from patients also confirmed the MMAF phenotype (**Figure 1B**). Altogether these results indicated that MMAF accompanies the underlying cause of infertility in these families.

### Whole-Exome-Identified Novel Loss-of-Function Mutations in *DNAH1*

In order to explore the underlying genetic cause of infertility, WES was performed on four patients, the control brother and their parents (III:2, IV:1, III:3, V:1, V:2, V:3, and V:4). Considering the consanguinity within the families, we screened the WES data and only retained the variants that displayed recessive inherence patterns. Furthermore, we filter the variants based on minor allele frequency, testis-specific expression, and review of literature about the genes that have an association with the MMAF phenotype. In short, we identified two novel mutations in *DNAH1*: one was the insertion of C (c.7646_7647InsC, p.N2549Qfs*61), causing a truncation of protein in the family A1, and the other was a missense mutation (c.6212T>G, p.C1789Y) in family A2 (**Figure 1D**). Sanger sequencing on DNA from all available members of both families indicated that the mutations are recessively segregating in all patients with infertility phenotype (**Figures 1Ca, b**). To check the effect of these mutations on protein function, we performed immunofluorescence (IF) of DNAH1 and alpha-tubulin on sperm smear slides from patients IV:1, V:1, and V:2. The IF result showed the absence of DNAH1 signals on the sperm tail of patients, while positive signals of DNAH1 were observed on the control sample (**Figure 1E**). Thus, these results indicated that the identified mutations are loss-of-function mutations and caused transcript decay by non-sense mRNA-mediated decay.

### Ultrastructural Analysis of Spermatozoa Displayed Numerous Defects in the Axonemal Structures

**Figure 2:**
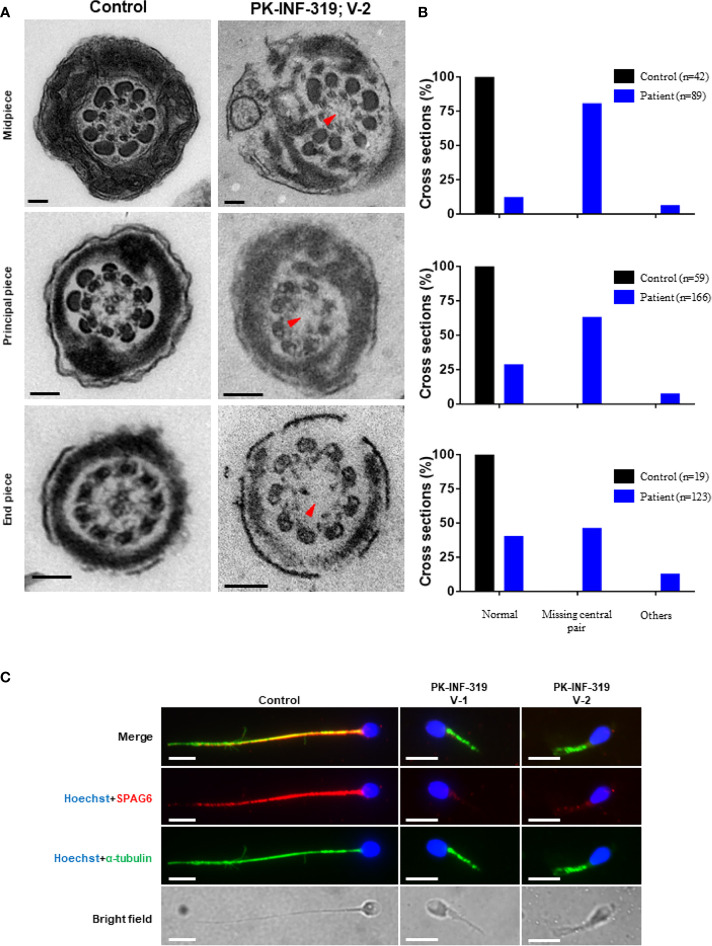
Transmission electron microscopic investigation of spermatozoa from patients and healthy control. **(A)** The cross-section of a healthy control spermatozoa represented an intact axoneme structure comprised of doublets of microtubules, circularly arranged around a central pair complex of microtubules (nine + two organization), surrounded by outer dense fibers and fibrous sheath. The cross-section of a patient spermatozoa displayed a missing central singlet of microtubules and disorganized fibrous sheath. Scale bars, 500 um. **(B)** Graphs representing the cross-sections of microtubule doublets corresponding to midpiece, principal piece, and end piece were statistically analyzed in control and patient. **(C)** The patient spermatozoa were devoid of SPAG6 signals (marker to check the integration of microtubules of the central pair), indicating disruption of inner dynein arm, while well-decorated SPAG6 signals were observed on the tail of spermatozoa obtained from a healthy control. Scale bars, 10 um.

In order to investigate the axonemal structures, we performed TEM on patient (IV:1) spermatozoa. We statistically analyzed good-quality cross-sections of microtubule doublets from the patient and control spermatozoa samples and found that the central singlet of microtubules was missing in 66% of these sections (**Figures 2A, B**). Furthermore, the fibrous sheath was disorganized in 10% of the sections, and missing microtubule doublets were observed in 6% of analyzed sections. To further confirm the disruption of the central pair, we stained the sperm smear slides of the patients with SPAG6 antibody, which is a well-known marker generally used to check the integration of microtubules of the central pair. No signals of SPAG6 were observed on the sperm flagella of the patients, while sperm from the control individual displayed well-decorated signals on the entire tail of the flagella (**Figure 2C**). Overall, these results intimidated that the identified mutations have a deleterious effect on the axonemal structures of sperm flagella.

### Generation of Mutant Mice Model

**Figure 3:**
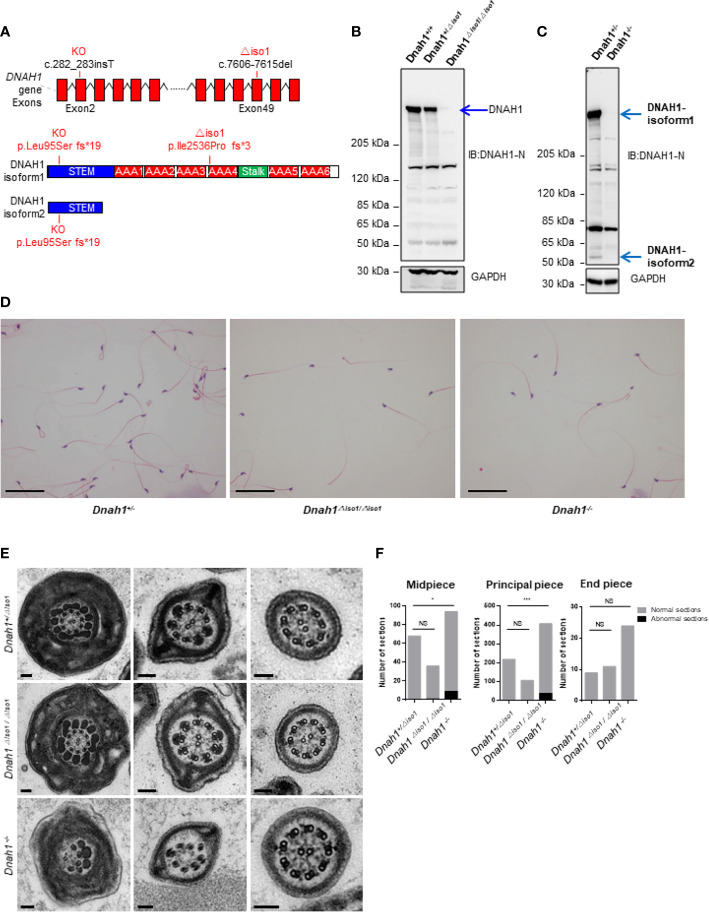
Generation of *Dnah1^-/-^* and *Dnah1^△iso1/△iso1^* mice. **(A)** Representative *Dnah1* structure and strategy used to generate *Dnah1^-/-^ and Dnah1^△iso1/△iso1^* mice through CRISPR/Cas9 genome editing technology by targeting exon 2 and exon 49, respectively. **(B, C)** Western blotting analysis confirmed the deletion of full-length DNAH1 protein in *Dnah1^-/-^* and *Dnah1^△iso1/△iso1^* mice testes, while isoform2 (50 kDa) still existed in *Dnah1^△iso1/△iso1^* mice testes. GAPDH was used as an internal control. **(D)** The sperm morphology analysis of *Dnah1^△iso1/△iso1^* mice displayed spermatozoa without a sperm tail. Scale bars, 10 um **(E)** Transmission electron microscopy investigation of *Dnah1^△iso1/△iso1^* mice spermatozoa showing an intact ultrastructure with the presence of a central pair and outer dense fibers, while abnormal sections were observed in *Dnah1^-/-^* mice spermatozoa. Scale bars, 10 um. **(F)** Quantification of normal and abnormal sections observed in the spermatozoa of *Dnah1^-/-^* and *Dnah1^△iso1/△iso1^* mice. *p < 0.05, ***p < 0.001 and NS, no significant difference.

In order to study the *in vivo* effects of mutations, we generated *Dnah1^-/-^* mice by deletion of the *Dnah1* transcript and *Dnah1^△iso/△iso1^* mutant mice similar to a mutation in family A2 (c.7646_7647InsC) by using CRISPR/Cas9 genome editing technology (**Figure 3A**). Sanger sequencing of the PCR products confirmed the genotype of *Dnah1^-/-^* and *Dnah1^△iso/△iso1^* mice (**Supplementary Figure S3A**). Furthermore, western blotting confirmed the absence of full-length DNAH1 protein in both mice models, while intact full-length DNAH1 (herein referred to as DNAH1 isoform1) protein was observed in *DNAH1^+/△iso1^* and *DNAH1^+/-^* mice testes (**Figure 3B**). Interestingly, we observed the presence of a short isoform of DNAH1 (referred to as isoform2) in the testes of *Dnah1^△iso1/△iso1^* mice, while no such isoform was found in *Dnah1^-/-^* mice (**Figure 3C**). Thus, it can be deduced that the *Dnah1^△iso1/△iso1^* mice still harbor the short-length DNAH1 protein. Similarly, reverse transcriptase PCR with a specific primer corresponding to isoform2 of DNAH1 also verified the presence of a small-length transcript in *Dnah1^△iso1/△iso1^* and wild-type (WT) mice (**Supplementary Figures S2A, B**). Moreover, we performed RT-PCR on control human spermatozoa to check the presence of isoform2. However, no isoform2 was detected in control spermatozoa, while a clear band corresponding to isoform1 was observed (**Supplementary Figure S2C**).

### Mutant Mice With Mild Ultrastructural Defects of Sperm Flagella

After confirming the deficiency of full-length DNAH1 protein in *Dnah1^△iso1/△iso1^* mice, we first scrutinized the fertility of mutant mice by caging one mutant mouse with two WT females for 3 months. During this duration, the female mice display vaginal plugs, demonstrating normal mating efficiency. However, no pregnancy was conceived, indicating that the *Dnah1^△iso1/△iso1^* mice were completely infertile (**Table 2**). Subsequently, testes morphology (**Supplementary Figure S3A**) and testes-to-body-weight ratio were comparable in mutant and WT mice (**Table 2**). On the other hand, reduced sperm count was evident in *Dnah1^-/-^* and *Dnah1^△iso1/△iso1^* mice (**Table 2**). Further examination of testis and epididymis section from *Dnah1^△iso1/△iso1^* mouse displayed an intact seminiferous tubule structure and the presence of all types of germ cells ranging from spermatogonia to spermatozoa (**Supplementary Figure S2C**). Similarly, an ample number of mature spermatozoa were present in the lumen of cauda in *Dnah1^△iso1/△iso1^* mouse, indicating that sperm production is not severely affected on the grass root level (**Supplementary Figure S3D**). Next, we analyzed sperm motility by CASA system and observed that progressive motility is severely compromised in *Dnah1^△iso1/△iso1^* mouse, while no progressive motile sperm was observed in *Dnah1^-/-^* mouse (**Table 2**). To explore the reason of reduced motility, we performed a comprehensive inspection of sperm morphology of *Dnah1^△iso1/△iso1^* mouse and *Dnah1^-/-^* mouse. Interestingly, we found that most of the spermatozoa (53/49%) lack full-length flagella structure, only possessing an intact head structure (**Figure 3D**), while few other abnormalities were observed (**Table 2**). Interestingly, there are still some (34/45%) sperm with normal flagella in *Dnah1^△iso1/△iso,^* and *Dnah1^-/-^* mouse, and the flagella without sperm head also have a normal morphology. On the other hand, ultrastructural investigation of sperm flagella from *Dnah1^△iso1/△iso1^* and *Dnah1^-/-^* mice displayed no significant variation in the architecture of axoneme (**Figures 3E, F**). However, statistical analysis indicated more abnormal sections in *Dnah1^-/-^* mouse than *Dnah1^△iso1/△iso1^* mouse, indicating that isoform2 may have a role in mediating the assembly and stabilization of axonemal structures. Altogether our study provided clues about the phenotype differences in *Dnah1* KO and *DNAH1* mutant human spermatozoa ultrastructure for the first time.

## Discussion

Sperm flagellum is a highly complex structure constituted by a series of proteins that mediate its assembly, composition, and function (41). Defects in sperm flagellum can occur by exogenous and endogenous factors, including genetic mutations that can also reduce sperm motility (42). Studies on mouse models have identified that several genes are required to properly develop the sperm flagellum. However, mutations in these genes are rarely reported in humans suffering from impaired motility due to the morphological defects of flagella. To date, variants in *AKAP4*, *CCDC39*, *DNAH1*, *CFAP43*, *CFAP44*, and *CFAP69* have been associated with MMAF phenotype in humans and mice (16, 27, 43–45). *DNAH1* is one of the most important members of the inner dynein arm and is considered an essential candidate gene for male infertility. The first mutation in *DNAH1* causing male infertility was reported in 2001 (26). Since then, various pathogenic mutations in *DNAH1* have been reported in various populations, causing male infertility (10, 11, 46, 47). However, most of the cases of altered sperm motility leading to human male infertility remain idiopathic. Thus, it is still difficult to identify the association between genetic mutations and impaired sperm motility.

In the current study, we recruited two extended consanguineous Pakistani families suffering from male infertility due to MMAF without having any symptoms of PCD. Next-generation sequencing identified two novel loss-of-function mutations in *DNAH1* (c.6212T>G, p.C1789Y, and c.7646_7647InsC, p.N2549Qfs*61) in family A1 and A2, respectively. These two variants were pathogenic in nature as no DNAH1 signals were observed on the sperm smear slides of the families of both patients, indicating that identified mutations caused the decay of mRNA transcript and the subsequent disruption of DNAH1 protein. Ultrastructural examination of patient spermatozoa revealed the absence of central singlet of microtubules, suggesting that mutations have adversely affected the axonemal structure. SPAG6 is an important marker that is widely used to check the integration microtubules of the central pair. No SPAG6 signals were observed on the patient spermatozoa, suggesting the disruption of the central pair. Subsequently, we generated mutant mice by targeting exon 49 of *Dnah1*, and western blotting confirmed the absence of a full-length DNAH1 protein. However, a short protein, possibly encoded by N-terminal (isoform2), was observed in the mutant and WT mice.

Interestingly, we observed mild defects in the ultrastructure of spermatozoa from mutant mice. We thought that maybe the isoform2 is mediating the assembly of microtubules in the absence of full-length DNAH1 protein in mice. Previous studies suggested that the N-terminal of the heavy chain of DNAH1 forms a stem structure and is required for the assembly dynein complex and cargo attachment (26, 48). In order to eliminate the effect of N-terminal-mediated isoform2, we generated *Dnah1^-/-^* mice by targeting exon 2 that completely disrupted the N-terminal, and western blotting confirmed the absence of full-length as well as isoform2 DNAH1 protein. Surprisingly, the TEM analysis of the ultrastructure of spermatozoa from *Dnah1^△iso1/△iso1^* and *Dnah1^-/-^* mice still showed no significant phenotype. Though more abnormal sections were observed in *Dnah1^-/-^* mouse as compared to *Dnah1^△iso1/△iso1^* mouse, *Dnah1^-/-^* mice spermatozoa still displayed intact central singlet of microtubules, suggesting that KO animals have some hidden factors that could ensure correct axonemal biogenesis and organization or that complete disruption of the heavy chain did not influence the assembly of the other components of an axonemal structure in mice. Alternatively, it can be hypothesized that the role of *DNAH1* in axonemal structure is not as central in mice as it is in humans.

Surprisingly, most of the spermatozoa from *Dnah1^△iso1/△iso1^* mouse and *Dnah1^-/-^* mice displayed an absence of the entire flagellum tail, a different phenotype from patients. The inner dynein arms are arranged in seven molecular complexes, viewed as globular heads arranged in three-two-two groups and corresponding to three different types of inner arms (IDA1 to IDA3). We assumed that one of the IDA has been disorganized in *Dnah1* mutant mouse spermatozoa, leading to loss of connection between external doublets of the microtubules and the two central microtubules and ultimately affected the cargo system required for cytoskeleton organization. Another possibility is that *Dnah1^△iso1/△iso1^* and *Dnah1^-/-^* spermatozoa are defective in organizing the α- and β-tubulins required for spermiogenesis (49). Thus, it can be inferred that *DNAH1* also regulates the α- and β-tubulin complexes, which play crucial roles in sperm tail elongation during spermiogenesis.

It is reasonable to hypothesize that the position of mutations in *DNAH1* may have a different effect on the sperm flagellum structure, which can completely reduce the immobility of spermatozoa. Furthermore, the mutations in the N-terminal and C-terminal of DNAH1 can display different effects on the axonemal structure of human spermatozoa. Altogether our study described that the role of DNAH1 is more central in regulating the axonemal assembly and dimerization in humans than in mice. The finding of this study will pave the way for finding out other intrinsic factors responsible for different phenotypes of DNAH1 mutations in humans and mice. Finally, it is suggested that screening the deleterious mutations of *DNAH1*could be important for the clinical molecular diagnosis of male infertility.

## Data Availability Statement

The original contributions presented in the study are included in the article/**Supplementary Material**. Further inquiries can be directed to the corresponding authors.

## Ethics Statement

The studies involving human participants were reviewed and approved by the Bioethical Committee of Pakistan and the ethical review board of the University of Science and Technology of China (USTC). The patients/participants provided their written informed consent to participate in this study. The animal study was reviewed and approved by the ethical review board of the USTC.

## Author Contributions

RK, JC, AM, and BZ performed the experiments. QZ, ManK, AA, MZ, WS, and MazK recruited the patients, performed semen analysis, and collected patient samples. MN gave insightful discussion and constructive comments on the manuscript. DZ and JZ performed the whole-exome sequencing (WES) and WES data analysis. QS, HZ, BX, YZ, and RK conceived and supervised the study, designed and analyzed the experiments, and wrote the manuscript. All authors contributed to the article and approved the submitted version.

## Funding

This work was supported by the National Natural Science Foundation of China (32070850) and National Key Research and Developmental Program of China (2018YFC1004700).

## Conflict of Interest

The authors declare that the research was conducted in the absence of any commercial or financial relationships that could be construed as a potential conflict of interest.

## Publisher’s Note

All claims expressed in this article are solely those of the authors and do not necessarily represent those of their affiliated organizations, or those of the publisher, the editors and the reviewers. Any product that may be evaluated in this article, or claim that may be made by its manufacturer, is not guaranteed or endorsed by the publisher.

## Supplementary Material

Supplementary Figure S1 Sperm morphology analysis. **(A)** Typical flagellar appearance in control subject. **(B)** Various defects of sperm flagella, such as absent, short, coiled, and bent, were common in the spermatozoa of patients.

Supplementary Figure S2 Presence of *DNAH1* isoform 2 in mutant and wild-type (WT) mice. **(A)** Gel electrophoresis of cDNA products (one, three, and five WT mice cDNA product; two, four, and six *Dnah1^△iso1/△iso1^* cDNA mice products) confirmed the presence of isoform 2 mRNA. **(B)** Corresponding Sanger sequencing of WT and *Dnah1^△iso1/△iso1^* mice cDNA further verified the presence of isoform 2 in *Dnah1^△iso1/△iso1^* mice. **(C)** Gel electrophoresis of cDNA product from control human spermatozoa confirmed the absence of isoform2 in humans.

Supplementary Figure S3 Genotyping and spermatogenesis in *Dnah1^-/-^* and *Dnah1^△iso1/△iso1^* mice. **(A)** Representative chromatograms from *Dnah1^+/-^*, *Dnah1^+/△iso1^*, *Dnah1^-/-^*, and *Dnah1^△iso1/△iso1^* mice confirming their genotype. The red letters indicate the changes in the DNA sequences. **(B)** Representative image of testes from adult rom *Dnah1^+/-^*, *Dnah1^+/△iso1^*, *Dnah1^-/-^*, and *Dnah1^△iso1/△iso1^* mice. Scale bars, 1 cm. **(C)** H&E staining of testes and caudal epididymis from adult *Dnah1^+/△iso1^* and *Dnah1^△iso1/△iso1^* mice. Scale bars, 50 μm.
