# Supplementary figures and images for: Novel Loss-of-Function Mutations in DNAH1 Displayed Different Phenotypic Spectrum in Humans and Mice
Source: Front Endocrinol (Lausanne). 2021 Nov 17;12:765639. doi: 10.3389/fendo.2021.765639 (PMC8635859; doi:10.3389/fendo.2021.765639)

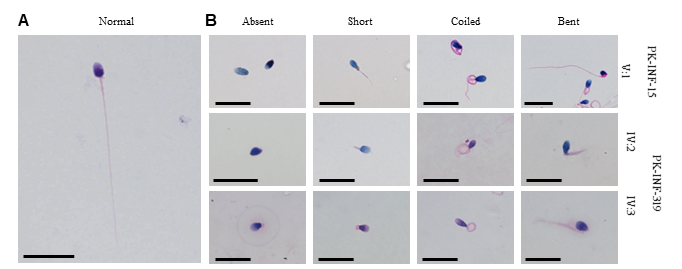

Supplement: Supplementary Figure S1 — Sperm morphology analysis. (A) Typical flagellar appearance in control subject. (B) Various defects of sperm flagella, such as absent, short, coiled, and bent, were common in the spermatozoa of patients. [file Image_1.tif]

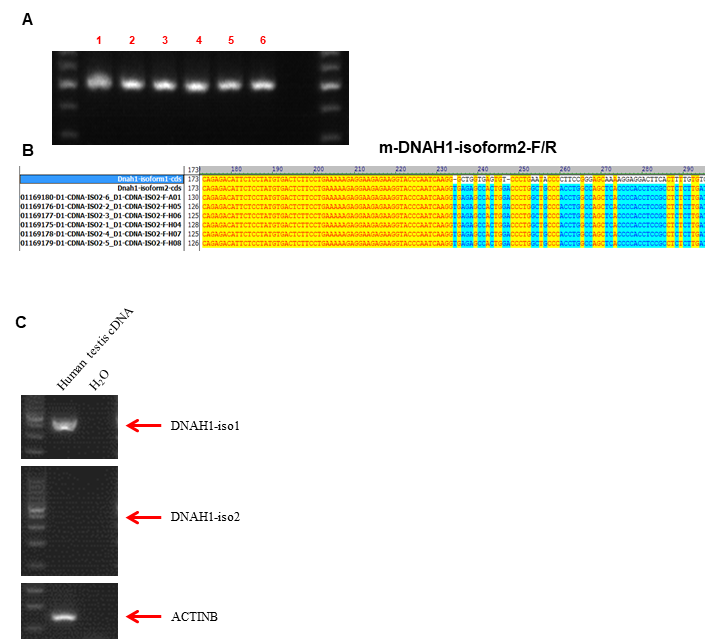

Supplement: Supplementary Figure S2 — Presence of DNAH1 isoform 2 in mutant and wild-type (WT) mice. (A) Gel electrophoresis of cDNA products (one, three, and five WT mice cDNA product; two, four, and six Dnah1△iso1/△iso1 cDNA mice products) confirmed the presence of isoform 2 mRNA. (B) Corresponding Sanger sequencing of WT and Dnah1△iso1/△iso1 mice cDNA further verified the presence of isoform 2 in Dnah1△iso1/△iso1 mice. (C) Gel electrophoresis of cDNA product from control human spermatozoa confirmed the absence of isoform2 in humans. [file Image_2.tif]

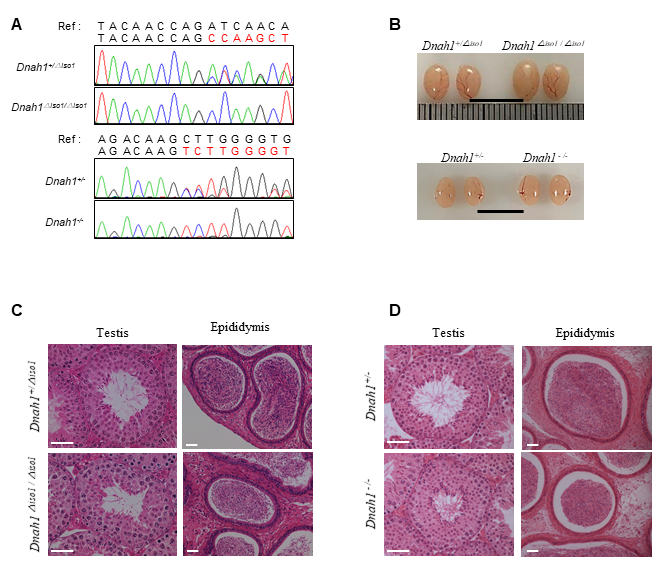

Supplement: Supplementary Figure S3 — Genotyping and spermatogenesis in Dnah1-/- and Dnah1△iso1/△iso1 mice. (A) Representative chromatograms from Dnah1+/- , Dnah1+/△iso1 , Dnah1-/- , and Dnah1△iso1/△iso1 mice confirming their genotype. The red letters indicate the changes in the DNA sequences. (B) Representative image of testes from adult rom Dnah1+/- , Dnah1+/△iso1 , Dnah1-/- , and Dnah1△iso1/△iso1 mice. Scale bars, 1 cm. (C) H&E staining of testes and caudal epididymis from adult Dnah1+/△iso1 and Dnah1△iso1/△iso1 mice. Scale bars, 50 μm. [file Image_3.tif]
