# Supplementary material for: Novel Loss-of-Function Mutations in DNAH1 Displayed Different Phenotypic Spectrum in Humans and Mice
Source: Front Endocrinol (Lausanne). 2021 Nov 17;12:765639. doi: 10.3389/fendo.2021.765639 (PMC8635859; doi:10.3389/fendo.2021.765639)
Supplement: Supplementary file 4 [file Table_1.docx]

| **Table S1. Primers used for mouse genotyping and RT-PCR** | | |
| --- | --- | --- |
| **Primers** | **Sequence** | **Product size (bp)** |
| *Dnah1^△iso1^* F | CACGGGAGAAAATCTGCAAC | 550 |
| *Dnah1^△iso1^* R | GAATCTCATGGACCGAGTAGG |  |
| *Dnah1^-/-^* F | GGTGTTAACTGGGCTGATTC | 548 |
| *Dnah1^-/-^* R | ATGGACCGAGGAATGGGATA |  |
| *mDnah1-cds-isoform2* F | GTCATCAATGAGCAGAGCTT | 490 |
| *mDnah1-cds-isoform2* R | CTTTTGACCATGGCTTTCCT |  |

| **Table S1. Primers used for *DNAH1* Sanger sequencing of patients and control siblings** | | |
| --- | --- | --- |
| **Primers** | **Sequence** | **Product size (bp)** |
| c.7646_7647InsC F | GGAATGTTCAGCTTGGGCTC | 442 |
| c.7646_7647InsC R | ATGGGTCAGTCAAACCGACC |  |
| *c.6212T>G* F | TGGCTTCACCAGCTACAGTC | 496 |
| *c.6212T>G* R | GACAGGTGGTCTTGTCCATG |  |
|  |  |  |
